# Leucine-Rich Diet Improved Muscle Function in Cachectic Walker 256 Tumour-Bearing Wistar Rats

**DOI:** 10.3390/cells10123272

**Published:** 2021-11-23

**Authors:** Laís Rosa Viana, Gabriela de Matuoka e Chiocchetti, Lucas Oroy, Willians Fernando Vieira, Estela Natacha Brandt Busanello, Ana Carolina Marques, Carla de Moraes Salgado, Alexandre Leite Rodrigues de Oliveira, André Schwambach Vieira, Paula Saenz Suarez, Lizandra Maia de Sousa, Bianca Gazieri Castelucci, Anibal Eugenio Vercesi, Sílvio Roberto Consonni, Maria Cristina Cintra Gomes-Marcondes

**Affiliations:** 1 Laboratory of Nutrition and Cancer, Department of Structural and Functional Biology, Institute of Biology, University of Campinas—UNICAMP, Campinas 13083-862, SP, Brazil; gamachi@unicamp.br (G.d.M.e.C.); loroy@etud.univ-angers.fr (L.O.); c104714@dac.unicamp.br (C.d.M.S.); 2 Polytech Angers, Biology and Health Systems Department, University of Angers, 49000 Angers, France; 3 Laboratory of Nerve Regeneration, Department of Structural and Functional Biology, University of Campinas—UNICAMP, Campinas 13083-862, SP, Brazil; williansfvieira@usp.br (W.F.V.); alroliv@unicamp.br (A.L.R.d.O.); 4 School of Medical Sciences, Department of Clinical Pathology, University of Campinas—UNICAMP, Campinas 13083-970, SP, Brazil; busanello.estela@gmail.com (E.N.B.B.); acarolinamarques@gmail.com (A.C.M.); anibal@unicamp.br (A.E.V.); 5 Laboratory of Electrophysiology, Neurobiology and Behaviour—LENC, Department of Structural and Functional Biology, Institute of Biology, University of Campinas—UNICAMP, Campinas 13083-862, SP, Brazil; asv@unicamp.br; 6 Laboratory of Cytochemistry and Immunocytochemistry, Department of Biochemistry and Tissue Biology, Institute of Biology, University of Campinas—UNICAMP, Campinas 13083-862, SP, Brazil; p179898@dac.unicamp.br (P.S.S.); l172421@dac.unicamp.br (L.M.d.S.); bgcast@unicamp.br (B.G.C.); consonni@unicamp.br (S.R.C.)

**Keywords:** cancer cachexia, leucine supplementation, muscle function, protein degradation

## Abstract

Skeletal muscle atrophy occurs in several pathological conditions, such as cancer, especially during cancer-induced cachexia. This condition is associated with increased morbidity and poor treatment response, decreased quality of life, and increased mortality in cancer patients. A leucine-rich diet could be used as a coadjutant therapy to prevent muscle atrophy in patients suffering from cancer cachexia. Besides muscle atrophy, muscle function loss is even more important to patient quality of life. Therefore, this study aimed to investigate the potential beneficial effects of leucine supplementation on whole-body functional/movement properties, as well as some markers of muscle breakdown and inflammatory status. Adult Wistar rats were randomly distributed into four experimental groups. Two groups were fed with a control diet (18% protein): Control (C) and Walker 256 tumour-bearing (W), and two other groups were fed with a leucine-rich diet (18% protein + 3% leucine): Leucine Control (L) and Leucine Walker 256 tumour-bearing (LW). A functional analysis (walking, behaviour, and strength tests) was performed before and after tumour inoculation. Cachexia parameters such as body weight loss, muscle and fat mass, pro-inflammatory cytokine profile, and molecular and morphological aspects of skeletal muscle were also determined. As expected, Walker 256 tumour growth led to muscle function decline, cachexia manifestation symptoms, muscle fibre cross-section area reduction, and classical muscle protein degradation pathway activation, with upregulation of FoxO1, MuRF-1, and 20S proteins. On the other hand, despite having no effect on the walking test, inflammation status or muscle oxidative capacity, the leucine-rich diet improved muscle strength and behaviour performance, maintained body weight, fat and muscle mass and decreased some protein degradation markers in Walker 256 tumour-bearing rats. Indeed, a leucine-rich diet alone could not completely revert cachexia but could potentially diminish muscle protein degradation, leading to better muscle functional performance in cancer cachexia.

## 1. Introduction

Skeletal muscle atrophy occurs in several pathological conditions such as cancer, especially during cancer cachexia. This condition is associated with a decrease in treatment response, reducing quality of life and increasing morbidity and mortality in cancer patients [1,2]. Cancer cachexia is a multifactorial syndrome consisting of involuntary body weight loss, especially by skeletal muscle and adipose tissue loss, reduced food intake, elevated resting energy expenditure, excess catabolism, and inflammation [3]. Skeletal muscle atrophy is the main problem of cancer cachexia due to its expressive contribution to total body composition. Skeletal muscle mass counts for up to 50% of total body protein in healthy individuals [4]. Striated skeletal muscle is an extremely adaptable tissue that can change structural and functional properties depending on the stimulation. The regulation of muscle mass is controlled by protein synthesis and degradation rates that should be balanced to maintain muscle mass [5]. Any change shifting the balance toward protein synthesis will lead to muscle hypertrophy, while changes shifting the balance toward protein degradation will lead to muscle atrophy. So far, three main pathways of skeletal muscle protein degradation have been identified: ubiquitin (Ub)-proteasome, cell autophagy/lysosomal and Ca^2+^-activated degradation pathways [6]. Among them, the Ub-proteasome system (UPS) is the main proteolytic machinery which is systematically activated in cachexia [7]. The activation of UPS pathways is often accompanied by the presence of inflammatory mediators, including IL-6 [8] and TNFα [9]. Moreover, the abnormal upregulation of muscle protein degradation is often related to the dysfunction of organelles, such as the endoplasmic reticulum (ER) [10] and mitochondria [11], which can lead to muscle function loss. Considering that skeletal muscle mass is the most representative tissue in our body, and muscle atrophy is a severe clinical problem related to poor prognosis and higher mortality, some studies have focused on the investigation of potential strategies—pharmacological and nonpharmacological—that could act as a coadjutant therapy to improve muscle mass and function. One of the nonpharmacological strategies is nutritional supplementation, and in this sense, leucine supplementation has a prominent place.

Leucine is an essential, anabolic, branched-chain amino acid that can promote muscle protein synthesis by increasing the activation of the mechanistic target of rapamycin (mTOR) [12]. Additionally, leucine supplementation also affects proteolysis by inhibiting relevant catabolic transcription factors, such as FoxO3 [13]. Considering this role of leucine, several preclinical studies of cancer cachexia have investigated the potential of leucine to diminish muscle atrophy as well as muscle molecular and metabolic alterations related to cachexia, attenuating muscle and body weight loss. Recent studies from our research group [14,15] have shown the molecular mechanisms of how leucine protects muscle from tumour atrophic factors in a cancer cachexia model, i.e., the Walker 256 carcinosarcoma. Cruz and colleagues [15] used both metabolomic and proteomic approaches to study the modulations of a leucine-rich diet in skeletal muscle during cancer cachexia. The authors showed a potential beneficial effect of leucine upon mitochondria, providing information about the muscle glycolytic pathways used by this amino acid [15]. This improvement may be associated with preserving muscle morphometric parameters and consequent protection against the effects of cachexia [15]. Also, the same research group [14] has shown that the leucine-rich diet modulated key steps of the synthesis (mTOR) pathway by triggering the increased activation of RAG and mTOR and maintaining JNK, STAT-3 and STAT-6 levels in muscle, leading to increased muscle protein synthesis, associated with lower degradation, minimising cancer-induced damages in the cachectic state. Before that, other research groups had investigated the potential role of leucine as a co-adjuvant treatment for cachexia associated with cancer. Peters and colleagues [16] performed a study on the effects of leucine supplementation on preserving muscle mass in C26 colorectal tumour-bearing mice, another widely used cancer cachexia experimental model. Despite not changing muscle atrophic markers such as atrogin and MuRF-1, leucine supplementation reduced muscle wasting and attenuated changes in plasma amino acids in tumour-bearing cachectic mice. Furthermore, using in vivo as well as in vitro models [17], supplementation with leucine or leucine metabolite β-hydroxy-β-methylbutyrate was shown to effectively diminish muscle atrophy, improving the molecular and metabolic alterations related to cachexia, and attenuating muscle and body weight loss.

The molecular mechanisms of how leucine protects muscle cells from degradation and atrophy are described; however, to date, no study has evaluated the effects of leucine via functional analyses, which are even more important from a translational perspective. Therefore, this study aimed to investigate the potential benefits of leucine supplementation through a functional assessment of the muscle by evaluating walking, general behaviour and strength, thereby contributing significantly to our current knowledge of the effects of leucine in cancer cachexia.

## 2. Materials and Methods

### 2.1. Experimental Design

**Figure 1:**
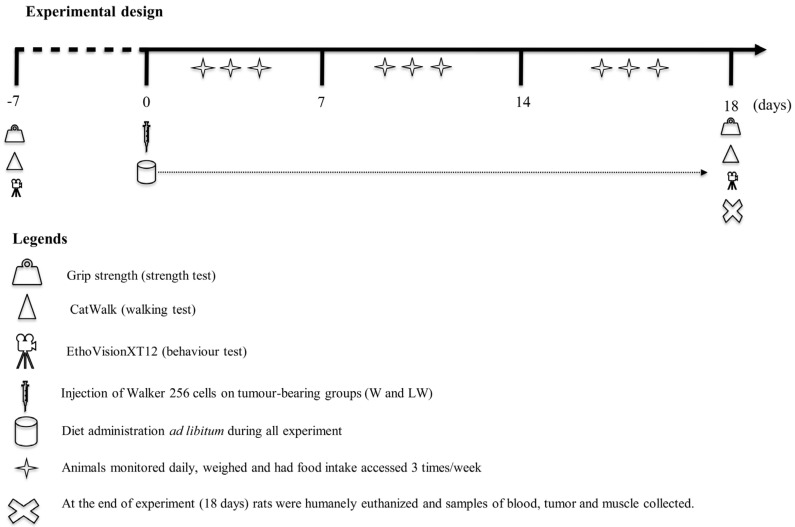
Experimental design. Male adult Wistar rats were monitored daily, weighed three times/week, and given food (control or leucine-rich diets) and water ad libitum. Food intake was measured three times/week, and the functional activities were accessed one week before (to access the health condition) and 18 days after tumour evolution and diet administration.

Male adult Wistar rats (approximately 12 weeks old, obtained from the Animal Facilities at the State University of Campinas, UNICAMP, São Paulo, Brazil) were housed in collective cages under controlled environmental conditions (light and dark 12/12 h; temperature 22 ± 2 °C; and humidity 50–60%), with ad libitum access to diet. Semipurified diets were prepared following the recommendations of the American Institute of Nutrition (AIN-93 [18]) and according to our previous studies [19,20,21]. The control diet contained 18% protein, and was composed of 20% casein (protein source), 39.7% corn starch, 13.2% dextrin, and 10% sugar (carbohydrate sources), 7% soy oil (fat source), 5% cellulose microfibre (fibre source), 3.5% salt mix, 1.0% vitamin mix, 0.3% cysteine, and 0.25% choline. The leucine-rich diet also contained 18% protein, and was composed of the same amounts of casein, fat, fibre, salt, vitamin mix, cysteine, and choline as the control diet. The addition of 3% leucine was followed by a 1% reduction in corn starch (38.7%), dextrin (12.2%), and sugar (9%). Within those adjustments, both diets, control and leucine, were normoproteic, isocaloric, and normolipidic. The widely used model of cancer cachexia, Walker 256 carcinosarcoma, was used in this study [22]. A cell suspension (2.5 × 10^6^ viable cells) of Walker 256 cells was injected subcutaneously into the right flank of the rats. The tumour inoculation and diet administration started on the same day (Figure 1).

The animals were randomly distributed into four experimental groups. Two groups were fed a control diet, i.e., Control (C) and Walker 256 tumour-bearing (W), while two other groups were fed a leucine-rich diet, i.e., Leucine Control (L) and Leucine Walker 256 tumour-bearing (LW). The minimal number of animals per group was six. The animals were monitored daily, weighed three times/week and given food and water ad libitum. Food intake was measured three times/week per week, and functional activities were assessed one week before (to assess the health condition) and 18 days after tumour and diet administration (endpoint moment) (Figure 1).

The final endpoint criteria utilised in the present study, the 18th day of tumour evolution, were determined based on the data derived from daily observations of discomfort symptoms such as piloerection, diarrhoea or constipation, hunched posture, tremors, closed eyes, and red tears (chromodacryorrhea). These symptoms were based on the indicators of quality of life proposed by Betancourt et al. [15]. At the endpoint moment (18 days following tumour inoculation), rats were killed by decapitation, and different body tissues, such as the spleen, perirenal fat, and skeletal muscle (musculus tibialis anterior, extensor digitorum longus (EDL) and soleus) were removed and weighed (Figure 1). The tibia length of each animal was used to normalise the corresponding tissues weights. EDL and soleus muscles were immediately placed on ice-cold buffer containing 10 mM Ca-EGTA buffer for oxygen consumption measurements. Muscle samples were frozen directly in liquid nitrogen and stored at −80 °C for further gene and protein expression analysis. Also, muscle fragments were also immediately fixed in 2.5% glutaraldehyde and 2.5% paraformaldehyde in sodium cacodylate buffer (0.1 M) at pH 7.4 and CaCl2 (3 mM) for 24 h at 4 °C before being processed for transmission electron microscopy analysis. Additional muscle samples were fixed in 4% paraformaldehyde for light microscopy assay. The general guidelines of the UKCCCR (United Kingdom Co-ordinating Committee on Cancer Research, 1998) [10] regarding animal welfare were followed, and the experimental protocol was approved by the Institutional Committee for Ethics in Animal Research (CEEA/IB/UNICAMP, protocol # 4289-1).

### 2.2. Muscle Functional Analysis

#### 2.2.1. Catwalk Walking Test

The catwalk walking test (Noldus Inc., Wageningen, The Netherlands) is an automated tool that quantifies gait parameters. Rats were placed in an illuminated-walkway glass floor with a video camera (Gevicam GP-3360; Gevicam Inc., Milpitas, CA, USA). The camera positioned under the walkway at a distance of 56 cm recorded the paws prints automatically by using the CatWalkTM XT10.6 software, as the animal crossed the pathway in a calibrated 20 × 10 cm length lane. The maximum intensity measurements were analysed, which represent the intensity of the full paw. The maximum contact area (cm^2^) corresponded to the max area of a paw that came into contact within the glass floor and the print area (cm^2^). The surface area of the complete paw print of both tumour-bearing groups (W and LW) was compared with the gait patterns at the initial time point (pretumour inoculation) and the endpoint moment (18 days after tumour inoculation). All experimental animals used were acclimated to the test one week before the experiment started. The software detects all paws during natural gait, recording the right front (RF), left front (LF), right hind (RH), and left hind (LH) paws. The average forepaws (RF and LF) were considered forelimb, and the average hind paws (RH and LH) were considered hindlimb. All data from tumour groups were compared to the initial time point (pretumour inoculation) and at the endpoint moment (~18 days after tumour inoculation).

#### 2.2.2. Behaviour Test (Video Recording System and Analysis)

Animal behaviour was assessed by night vision cameras placed in front of each individual cage at an adequate height. The cameras recorded all rats’ nocturnal behaviour. The video recording system was activated one week before the experiment started. Video files were analysed using the video tracking software EthoVisionXT12 (Noldus Information Technology, Utrecht, The Netherlands) to assess the total distance covered (cm), the average velocity (cm/s) and the time spent in movement (s) of both tumour-bearing groups (W and LW). All tumour-group data were compared to behaviour at the initial time point (pretumour inoculation) and at the endpoint moment (18 days after tumour inoculation).

#### 2.2.3. Grip Strength Test

A grip strength test was performed randomly in all experimental groups at the beginning and endpoint of the experiment to take force measurements. The equipment use procedures followed the manufacturers’ instructions (BIOSEB’s Grip Strength Test) and were always undertaken by the same researcher during the morning period. The animals were placed in the grip strength room 15 min before the test to acclimate them to the environment. Briefly, rats were held by the tail and lowered towards the grip strength meter. The animals were allowed to grab the metal grip, and were then pulled backwards on the horizontal plane. The force applied to the grid just before the animal lost grip was recorded as the peak tension. Measurements were repeated 10 times for each animal, and were recorded in grams and then normalised according to the tibia length of each animal.

### 2.3. Skeletal Muscle Tissue Sample Preparation for Oxygen Consumption

Oxygen consumption was determined according to a previous study performed by Busanello and colleagues [23]. Briefly, soleus and extensor digitorum longus (EDL) muscles were harvested from Walker 256 tumour-bearing Wistar rats from W and LW groups and placed on ice-cold buffer containing 10 mM Ca-ethylene glycol-bis (B-aminoethyl ether)-N′N′N′N′-tetraacetic acid (EGTA) buffer (2.77 mM of CaK_2_EGTA + 7.23 mM of K_2_EGTA, free concentration of calcium 0.1 mmol/L), 20 mmol/L imidazole, 50 mmol/L K +/4-morpholinoethanesulfonic acid, 0.5 mmol/L dithiothreitol, 7 mmol/L MgCl_2_, 5 mmol/L ATP, 15 mmol/L phosphocreatine, pH 7.1. Individual bundles from eight to eleven mg of muscles tissue were separated with forceps. Samples were permeabilised in ice-cold buffer containing saponin (50 μg/mL) for 30 min, gently stirred, and washed three times with MiR05 medium (60 mmol/L potassium lactobionate, 0.5 mmol/L EGTA, 3 mmol/L MgCl_2_, 20 mmol/L taurine, 10 mmol/L KH_2_PO_4_, 20 mmol/L HEPES, 110 mmol/L sucrose, 1 g/L bovine serum albumin [BSA], pH 7.1) at 4 °C. Samples were dried with filter paper and weighed [24].

Permeabilised tissues were added to a MiR05 medium containing EGTA (500 mM) at 37 °C supplemented with 10 mM glutamate plus 5 mM malate in an Oroboros oxygraph (Innsbruck, Austria). ADP (400 μM), oligomycin (0.63 μM), and FCCP (0.6 μM) were added during the experiments.

### 2.4. Muscle Morphological Analyses

#### 2.4.1. Light Microscopy

Muscle samples were removed from animals and immersed in a fixative solution (4% paraformaldehyde in 0.1 M phosphate-buffered saline (PBS), pH 7.4) for 24 h at 4 °C. Then, tissues were dehydrated in graded alcohol concentrations, embedded in paraffin (Leica Microsystems, Heidelberg, Germany), and sectioned at a width of 3 μm. The sections were mounted on slides and stained with hematoxylin-eosin. A cross-sectional area of myofibre was measured for each muscle sample using at least 200 fibres per muscle sample (average number ± SD of fibre analyses for each group). Analyses were performed using Image Pro-Plus Premium software (v.3.01, Media Cybernetics, Silver Spring, MD, USA) after capturing the image with a Leica microscope (Leica DMLM, Wetzlar, Germany) using 20× magnification.

#### 2.4.2. Transmission Electron Microscopy

A transmission electron microscope was used as follows: the skeletal muscle tissue was immersed and fixed in a solution consisting of 2.5% glutaraldehyde and 2.5% paraformaldehyde in sodium cacodylate buffer (0.1 M) at pH 7.4 and CaCl_2_ (3 mM) for 24 h at 4 °C. Then, tissue samples were rinsed with cacodylate buffer/CaCl_2_ and were post-fixed in 1% OsO_4_ in sodium cacodylate buffer (0.1 M), CaCl_2_ (3 mM), and potassium ferrocyanide solution (0.8%) for 1 h on ice. Following, tissue samples were washed with Milli-Q water and stained with uranyl acetate (2%) overnight at 4 °C. Then, tissue samples were washed in milli-Q water and dehydrated in an ethanol gradient. The samples were embedded in Epon 812 resin. Resin polymerisation was controlled in an incubator (60 °C) for 72 h. Ultra-thin sections were stained with uranyl acetate and lead citrate, then observed in a transmission electron microscope LEO 906 (Zeiss, Oberkochen, Germany), operated at 60 kV.

### 2.5. Serum and Muscle Molecular Analyses

#### 2.5.1. Serum Cytokines Assay

The serum cytokine (IL-6 and TNF-α) profile was measured by Luminex assay using a specific kit (Rat Premixed Multi-Analyte kit) from R&D System^®^ (Minneapolis, MN, USA) following the manufacturer’s technical procedures.

#### 2.5.2. Quantitative RT-PCR 

Total RNA from the tibialis anterior muscle tissue was extracted with TRIZOL^®^ reagent (Invitrogen) following the manufacturer’s instructions. The quality of the RNA samples was examined at 260/280 nm and 260/230 nm with a UV spectrophotometer (Nanovue Spectrophotometer 28923215 Ge BioSciences, Chicago, IL, USA). cDNA was produced using a high capacity cDNA reverse transcription kit (Applied Biosystems^®^, Waltham, MA, USA) containing Multiscribetm Reverse Transcriptase. cDNA synthesis was performed on 1 μg of RNA at 42 °C. Real-time reactions were performed using standard methods (ABI Prism 7500 Sequence Detection System; Applied Biosystems, Foster City, CA, USA), and qPCR analysis was normalised to GAPDH. The genes evaluated using qPCR were FoxO3 (forward primer 5′- AACTTTGAC TCC CTC ATC TC -3′ and reverse primer 5′- TTT TCT CTG TAG GTC TTC GG -3′), IL-6 (forward primer 5′- ACT CAT CTT GAA AGC ACT TG -3′ and reverse primer 5′- GTC CAC AAA CTG ATA TGC TTA G -3′), ubiquitin (forward primer 5′- CAA GCT CAG TCT TTT GCC TCA GA -3′ and reverse primer 5′- GGA TCG GCG GGT AAT GAA G -3′), COX5a (forward primer 5′-TGTTGGCTATGATCTGGTTCC-3′ and reverse primer 5′-TTATGAGGTCCTGCTTTGTCC-3′), CS (forward primer 5′-TATGGCATGACGGAGATGAA-3′ and reverse primer 5′-CATGGACTTGGGCCTTTCTA-3′), and GAPDH forward primer 5′- CCA TGG AGA AGG CTG GG -3′ and reverse primer 5′- CAA AGT TGT CAT GGA TGA CC -3′).

#### 2.5.3. Western Blotting

Samples of tibialis anterior muscle biopsies were lysed in RIPA buffer (150 mM NaCl, 25 mM Tris-Cl, pH 7,4, 0.1% SDS, 1% NP-40, 0.5% sodium deoxycholate) and supplemented with protease and phosphatase inhibitors. Following the protein extraction protocol, protein concentration was measured using the bicinchoninic acid (BCA) method, according to the manufacturer’s instructions (Pierce™ BCA Protein Assay Kit, Sigma Aldrich, Poole, UK). The proteins (40 μg) were separated by electrophoresis, transferred to nitrocellulose membranes, and stained proteins with Ponceau S. After that, the membranes were incubated with primary antibodies against FoxO1 (2880) (Cell Signalling, Danvers, MA, USA), MuRF1(SC32920) (Santa Cruz Biotechnology, Santa Cruz, CA, USA), 20S (PW8195) (Enzo Life Sciences, Farmingdale, NY, USA), and GAPDH (SC47724) (Santa Cruz Biotechnology, Santa Cruz, CA, USA) as a loading control. Later, the membranes were probed with secondary antibodies conjugated with peroxidase (secondary antibodies goat antirabbit (7074) and horse antimouse (7076) (Cell Signalling)), and bands were visualised using a chemiluminescent reagent (Thermo Fisher Scientific, Waltham, MA, USA). The membrane images were captured using an imaging system (Amersham Imager 600, GE Healthcare), and band volume quantitation was quantified.

### 2.6. Statistical Analysis

Data are expressed as mean ± SEM. Differences between three or more groups were analysed by variance test ANOVA, followed by Tukey post hoc test and by *t*-test for comparison between 2 groups (W vs. LW). For all statistical analyses, *p* < 0.05 was considered significant. The statistical analyses were performed using the software Graph Pad Prism 9.0 (Graph-Pad Software, Inc., San Diego, CA, USA). All comparisons among the four groups processed by ANOVA were presented as Supplementary Figures S1–S6. The result section only presented the comparison between LW vs. W, showing the specific leucine effect.

## 3. Results

### 3.1. Leucine-Rich Diet Had No Effect in the Walking Test but Improved Muscle Strength and General Behaviour

**Figure 2:**
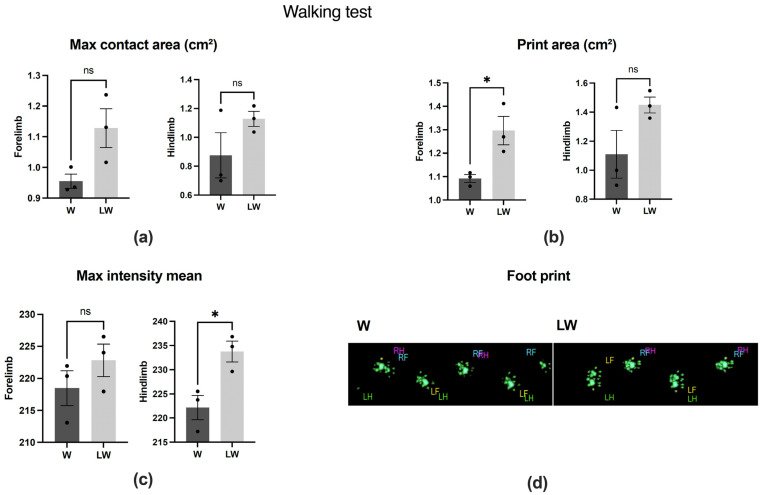
Catwalk functional (Walking test) parameters analyses in tumour-bearing groups, fed or not the leucine-rich diet. (**a**): Maximum contact area (cm^2^) in forelimb and hindlimb paws; (**b**): Print area (cm^2^) in forelimb and hindlimb paws; (**c**): Maximum intensity mean in forelimb and hindlimb paws and (**d**): A representative image of 2D walking pattern of both W and LW groups after (endpoint) tumour inoculation. (*N* = 3). For details, see the Methods section. RF—right front; LF—left front; RH—right hind; LH—left hind. The average forepaws (RF and LF) was considered forelimb, and the average hind paws (RH and LH) were considered hindlimb. Graphics represent mean ± SEM. * *p* < 0.05 and ns = not significant by *t*-test.

**Figure 3:**
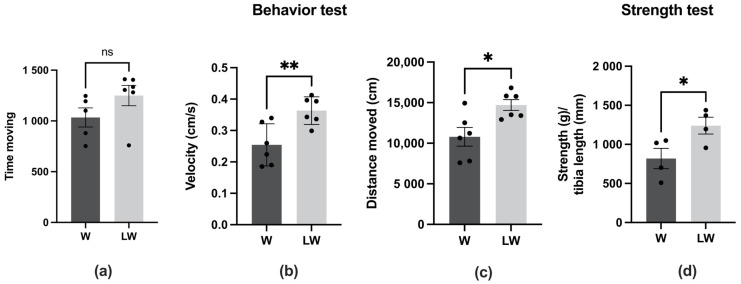
Evolution of behaviour and muscle force in tumour-bearing groups, fed or not the leucine-rich diet. Ecthovision behaviour parameters measured at night time. (**a**) Time moving, (**b**) Velocity and (**c**) Distance moved measured at endpoint moment. *N* = 6 animals per group. * *p* < 0.05, ** *p* < 0.01, and ns = not significant by *t*-test. Strength test measurement. (**d**): Strength (g) normalised by tibia length (mm) measured at endpoint moment, *N* = 4 animals per group. For details, see the Methods section. Graphics represent mean ± SEM. * *p* < 0.05, ** *p* < 0.01 and ns = not significant by *t*-test.

As expected, the cachexia state resulting from Walker 256 tumour growth led to impaired muscle function, which was assessed in this present study by walking, behavioural, and strength tests (Supplementary Figures S1 and S2). In order to assess the effect of leucine supplementation in cancer cachexia groups, we compared W and LW groups after tumour inoculation. The print area of the forelimb and the maximum intensity mean of the hindlimb were positively improved by the leucine-rich diet (LW > W; Figure 2b,c); however, for the other walking test analysed parameters, leucine had no effect (LW = W) (Figure 2a,b). Similarly, leucine did not affect the time moving (Figure 3a) evaluated by the behaviour test.

On the other hand, the other two parameters analysed by general behaviour, i.e., velocity and distance moved, were improved in tumour-bearing groups fed with leucine (LW) in comparison to tumour control group (LW > W; Figure 3b,c). In agreement with the preservation of velocity and total distance moved, the force measurement, determined by grip strength, was higher in LW than in the W group (Figure 3d).

### 3.2. Leucine-Rich Diet Improved Body, Fat, and Muscle Mass but Did Not Prevent Inflammation of Cachectic Tumour-Bearing Rats

**Figure 4:**
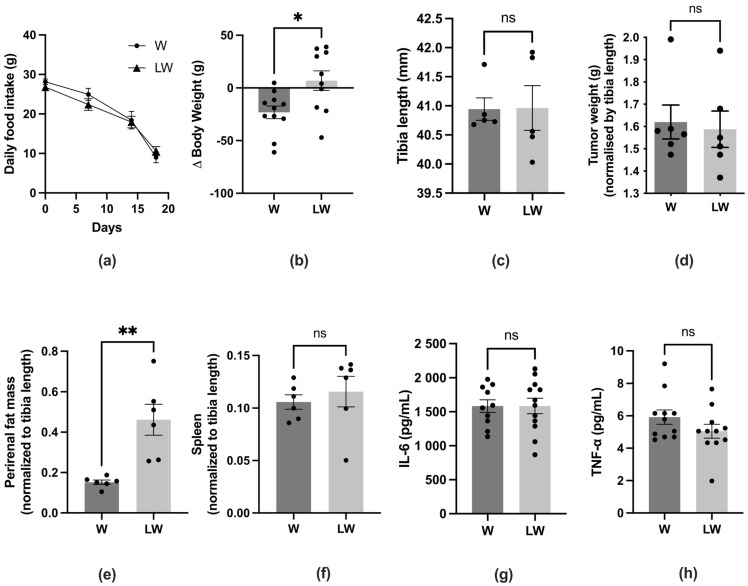
Morphological parameters. (**a**) Daily food intake (g) during the experimentation protocol; (**b**). Delta body weight (g) ((final body weight-tumour weight)-initial body weight); (**c**) Tibia length (mm); (**d**) Tumour weight (normalised by the respective tibia length); (**e**) White adipose tissue mass (perirenal) (normalised by the respective tibia length); (**f**) Spleen mass (normalised by the respective tibia length); (**g**) Serum IL6 concentration (pg/mL); (**h**) Serum TNF-α concentration (pg/mL). For details, see the Methods section. *N* = minimum of 6 animals per group. Graphics represent mean ± SEM. * *p* < 0.05, ** *p* < 0.01, and ns = not significant by *t*-test.

**Figure 5:**
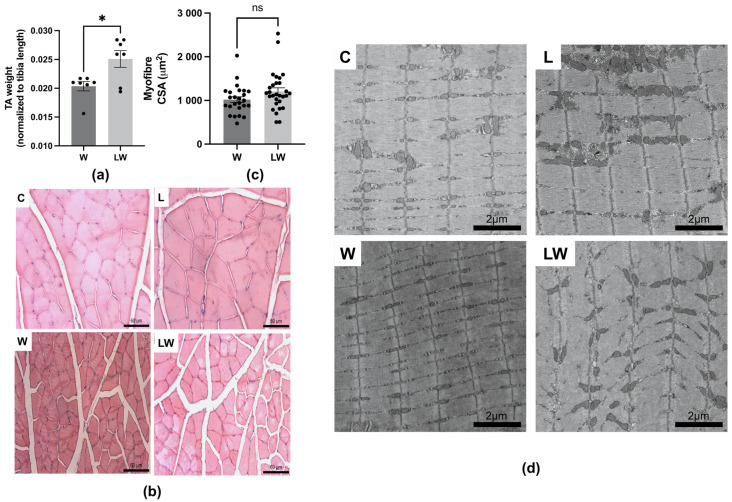
Morphometric, histological and ultrastructural analyses of tibialis anterior muscle among the different experimental groups. (**a**) Tibialis anterior (TA) muscle mass (normalised by the respective tibia length). (**b**) Representative light microscopy of muscle tissue cross-section, stained by Hematoxylin Eosin, in C, L, W and LW groups (Scale bar: 60 μm. Magnification 40×). (**c**) Measurements of myofibre cross-sectional area (μm^2^). (**d**) Representative transmission electron microscopy of myofibre showing sarcomeric arrangement and mitochondria distribution at cytoplasm in C, L, W and LW groups (Scale bar: 2 μm. Magnification at 10,000×). For details, see the Methods section. *N* = 6 animals per group. Graphics represent mean ± SEM. * *p* < 0.05 and ns = not significant by *t*-test.

The muscle function decline in Walker 256 tumour-bearing rats was accompanied by typical cachexia symptoms observed in cancer-cachectic hosts (Supplementary Figure S3). Despite the fact that the initial body weight was similar among all groups (Supplementary Figure S4a), the food intake (Figure 4a) and the delta body weight (Figure 4b—represented by the difference between final body weight without tumour mass and initial weight) were reduced in W and LW groups after tumour evolution. Despite also being decreased in the LW group compared to nontumour groups, the delta body weight in LW was still positive and significantly improved in comparison to the W group (Figure 4b). The tibia length, a parameter that remained unchanged even with body mass loss, was measured in each animal (Figure 4c) to normalise the tissues mass. Even with similar tumour mass (Figure 4d), the tibialis anterior muscle and perirenal fat mass were significantly higher in LW than the W group (Figure 4e and Figure 5a). Despite showing a beneficial effect in maintaining body weight, fat, and muscle mass, the leucine-rich diet could not prevent the inflammatory state imposed by cancer cachexia. In this way, we observed a higher spleen mass (LW = W; Figure 4f) and increased serum content of the pro-inflammatory cytokines IL-6 and TNF-α, which was not significantly different between both tumour-bearing groups (LW = W; Figure 4g,h).

### 3.3. Despite Preserving Muscle Mass, Leucine-Rich Diet Had No Effect on Muscle Microscopic Structures

As a consequence of cachexia, the tibialis anterior muscle mass reduced in the W group in relation to C group (Supplementary Figure S4a), but between both tumour-bearing groups, muscle mass was significantly higher in LW than the W group (Figure 5a). On the other hand, the leucine-rich diet had no effect on myofibre cross-sectional area (CSA) (LW = W; Figure 5b,c). A transmission electron microscopy analysis revealed the ultrastructural features in all groups (Figure 5d), showing that the sarcomeric arrangement, sarcoplasmic reticulum, T tubules, and mitochondria through the cytoplasm apparently had no significant modification in all groups. However, the sarcomere structure diameter at the W group seemed thinner than those of the other groups, which was consistent with the muscle atrophy compared to C group. Morphological observations led us to infer that these alterations might be more functional than structural at the tibialis anterior muscle. Although leucine supplementation had no effect on muscle atrophy (CSA), it increased the muscle total protein concentration (Supplementary Figure S7c).

### 3.4. Leucine-Rich Diet Decreased the Expression of MuRF-1 and 20S Proteins, but Had No Effect on Oxidative Capacity

**Figure 6:**
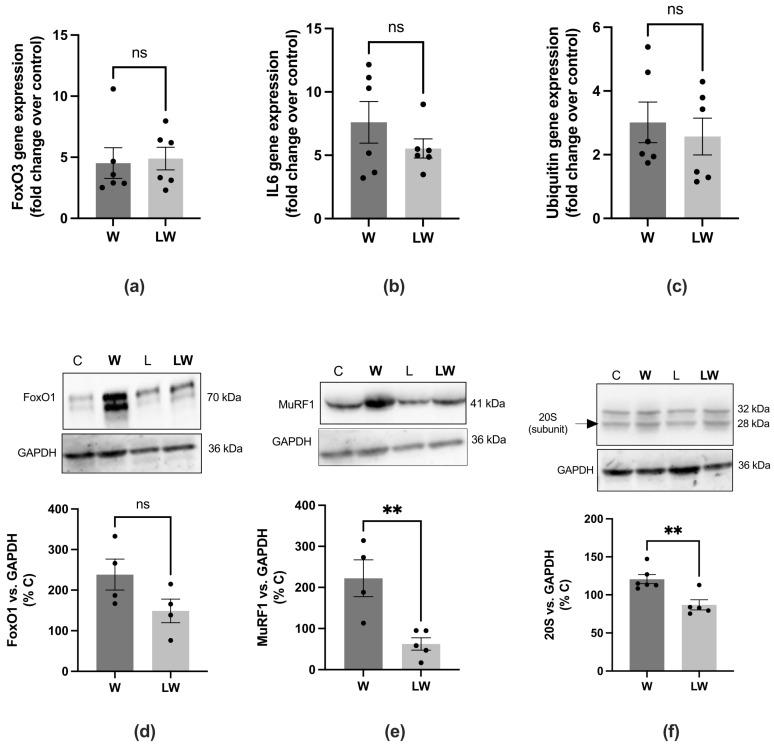
Muscular gene and protein expression among the different experimental groups. Gene expression of (**a**) FoxO3, (**b**) IL6 and (**c**) ubiquitin. Protein expression. Western blot analysis images from (**d**) FoxO1, (**e**) MuRF-1, and (**f**) 20S expressions in tibialis anterior muscle biopsies. Bar graphs indicating western blot analysis representing values of band volume. GAPDH was the housekeeping protein. For details, see the Methods section. Graphics represent mean ± SEM. *N* = 6 animals per group.** *p* < 0.01 and ns = not significant by *t*-test.

It is also well established that protein degradation is upregulated in cachectic muscle. We evaluated the expression of key genes related to muscle catabolism, and found that the expression of the FoxO3, IL-6, and ubiquitin genes was increased in both the W and LW groups (Figure 6a–c). Although the increase in catabolic genes was evident independent of leucine supplementation, the protein expression of MuRF-1 and proteasome 20S subunit were significantly decreased in LW in comparison to the W group (Figure 6e,f).

**Figure 7:**
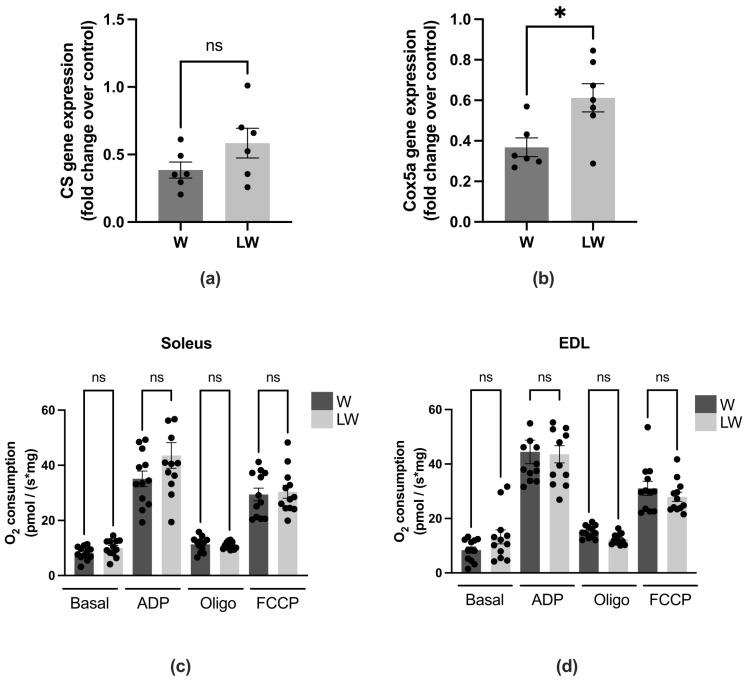
Muscular gene and protein expression among the different experimental groups. (**a**) Gene expression of citrate synthase and (**b**) (CS) cyclooxygenase (COX)5a. O_2_ consumption (ρmol O_2_/s. mg tissue) compiled from the respiration traces comparing the (**c**) soleus and (**d**) EDL muscles biopsies from both tumour-bearing rats (W vs. LW group). For details, see the Methods section. For gene expression *N* = 6 and for oxygen consumption *N* = 10–12, at least ten independent experiments. Graphics represent mean ± SEM. * *p* < 0.05 and ns = not significant by ANOVA followed by *t*-test.

Besides the protein degradation pathway, the muscle mitochondrial oxidative metabolism was also investigated. The gene expression of citrate synthase (CS), related to oxidative metabolism, was similar between tumour-bearing groups (LW = W) (Figure 7a), and Cox5a gene expression, which is important to maintaining the normal mitochondrial function, was higher in LW in comparison to W group (Figure 7b). In order to determine if this increase in Cox5a gene expression would result in a greater OXPHOS function, we measured oxygen consumption from two different metabolic muscles, soleus (which has a greater proportion of type I fibres, oxidative) and EDL (which has a greater proportion of type II fibres, glycolytic) [25]. Despite the greater Cox5a gene expression in LW muscle compared to the W group, oxygen consumption did not change between the W and LW groups. The mitochondrial respiration rates of both soleus (Figure 7c) and EDL (Figure 7d) muscles in all measured conditions, phosphorylating (ADP), resting (oligomycin), and maximal (FCCP), were the same between both tumour-bearing groups (LW = W) (Figure 7c,d). Corroborating with these results, the protein expression of OXPHOS complexes (CI-CV) was not altered in muscle tissue from tumour-bearing groups (LW = W) (data not shown).

## 4. Discussion

Cancer cachexia is characterised by a significant involuntary body weight loss, mainly related to skeletal muscle loss [3,26]. Muscle atrophy is induced by tumour and host-released factors that lead to a chronic inflammatory state, activating proteolysis and inhibiting protein synthesis. In addition, the cachectic patient also presents a significant reduction in muscle function, which is related to reduced quality of life. In this context, some nonpharmacological strategies, such as leucine supplementation, have been studied as potential co-adjuvants in cancer treatment targeting the maintenance of muscle mass. Although some molecular mechanisms of how leucine protect muscle cells from Walker 256 tumour evolution have already been described, no study so far has evaluated the effects of leucine within functional analyses, which is even more important from a translational perspective. Therefore, this study evaluated the effects of a leucine-rich diet on muscle function activity of cachectic Walker 256 tumour-bearing rats and correlated such effects with the molecular pathways of muscle atrophy.

As expected, Walker 256 tumour growth impaired muscle function, as shown in walking, behaviour, and strength tests, as well as morphometric parameters such as body weight and skeletal muscle loss. It is important to highlight that tumour growth was accompanied by a drastic (~70%) reduction in food intake, which may have led to some of the metabolic modifications observed in this study. As the aim of this study was to assess the effect of leucine supplementation in tumour-bearing animals, we compared the results only from both cancer cachexia groups. In this context, we found that the reduction of food intake between tumour groups was the same, so we can attribute the obtained results to the leucine effect. Our results showed that a leucine-rich diet improved muscle strength and general behaviour (maintained the velocity and distance moved). The positive effects of leucine in muscle function have also been observed in other muscle atrophy conditions such as ageing and disuse. In a clinical study performed by Martinez and colleagues [27], the administration of leucine significantly improved some functional performance parameters, e.g., walking time and lean mass index in sarcopenic elderly individuals. Furthermore, the leucine-treated group showed significantly improved respiratory muscle function, measured by the maximum static expiratory force, compared to placebo. The authors concluded that leucine supplementation can have some beneficial effects on sarcopenia, and could be considered for the treatment in older individuals [27]. Another study found that leucine partially protects muscle health, i.e., metabolism, mass, and function, during relatively brief periods of physical inactivity in middle-aged adults [28]. In the present study, we found that the group fed with leucine supplementation (LW) presented better muscle strength, general behaviour, and greater body, muscle, and fat mass. However, no effect was observed in the walking test, inflammation, and OXPHOS function.

A study performed by VanderVeen and colleagues [29] showed that a slow-fatigable contractile phenotype is developed during the progression of cachexia, and that this is directly related to increased muscle inflammatory signalling. According to this study, we also found a decrement in skeletal muscle function during cachexia evolution that was highly impacted in the advanced stages of cachexia, accompanied by smaller myofibre size and upregulated inflammatory signalling. As presented here, although muscle mass was bigger in the leucine-treated group, no difference was found in myofibre size (CSA) and ultrastructure analyses. The fact that the increase in muscle mass was not accompanied by an increase in muscle CSA could be partially explained by the enhanced content of total muscle protein found in leucine treated group. Moreover, the heavier muscle in the leucine group may be related to a greater muscle fat deposit. It is known that leucine is capable of regulating lipid metabolism by lipid metabolism-related genes [30]. A study from Zampierie and colleagues evaluated the effects of leucine supplementation in an obesity model, and found that fat mass was increased in leucine-supplemented rats. The authors also observed that leucine produced a hypothalamic pattern of gene expression that favoured fat accumulation [31]. Although the authors did not evaluate fat content in muscle, we can hypothesise that leucine may also lead to an intermuscular adipose deposition. It is very well established that skeletal muscle constitutes an important site for lipid utilisation. Considering this point, the study by Sun and Zemel (2007) demonstrated that leucine participates in the regulation of fatty acid oxidation in skeletal muscle cells in vitro, with leucine promoting fatty acid oxidation [32]. Although increasing lipid supply to skeletal muscle is not always beneficial, it is highly dependent on the biological context [33]. Possibly, in the context of cachexia, it may serve as an additional energy source for muscle, thereby benefiting muscle function. Knowing that the proteolytic pathway is highly expressed in cachectic muscle and that its activation leads to muscle atrophy, we hypothesise that a leucine-rich diet decreases, even partially, the activation of proteolytic/catabolic pathways. Here, we found that even with increased IL-6 and FoxO3 expression in both tumour-bearing groups, leucine supplementation blunted the expression of key catabolic related proteins, MuRF-1, and 20S. It is also known that leucine enhances muscle protein synthesis [34,35,36]. However, in the present study, the main protein synthesis pathway, e.g., mTOR, was not altered in cachectic Walker 256 tumour-bearing rats, so a leucine-rich diet did not affect this pathway (data not shown).

Interestingly, in addition to genes linked with proteolytic/catabolic pathways, more than 10% of atrophy-related genes are directly involved in energy production. Furthermore, several genes coding for essential glycolysis and oxidative phosphorylation enzymes are coordinately suppressed in atrophying muscles [37]. These points suggest that alterations in mitochondria and the morphology of the mitochondrial network can have potentially deleterious consequences in terms of muscle mass and function stability. A study performed by Fontes-Oliveira and colleagues [11] found that cancer cachectic muscle undergoes profound morphological changes, which are visualised mainly in alterations in sarcoplasmic reticulum and mitochondria. These alterations are linked to pathways that can account for inefficient energy sources associated with cancer cachexia. Our initial hypothesis was that leucine would improve OXPHOS capacity and energy production due to the fact that acetyl-CoA, which is one of the final catabolic products of leucine metabolism, can be directly consumed by mitochondria through the TCA cycle [38], favouring OXPHOS. To investigate this point, we verified the mitochondrial function-associated gene expression of Cox5a, and citrate synthase, directly related to oxidative metabolism in the muscle of cancer cachexia groups. We found that leucine supplementation led to increased Cox5a gene expression, even though oxygen consumption did not change between both tumour-bearing groups. Corroborating these results, the protein expression of OXPHOS complexes (CI-CV) was not altered. Although we did not find any effect of leucine in enhancing oxidative capacity in cancer cachexia animals, Vaughan and colleagues [39] evaluated the effects of leucine treatment on oxidative and glycolytic metabolism in humans and murine skeletal muscle cells. The authors also observed a significant reduction in glycolytic metabolism and on lactate export in leucine-treated cells. Therefore, Vaughan and colleagues concluded that leucine could potentially induce an oxidative profile by increasing the oxidative capacity in skeletal muscle cells [39]. Furthermore, a study performed by Pereira and colleagues (2015) found that leucine decreased the inflammation area and induced an increase in the number of proliferating satellite cells in muscles [40]. Taken together, we may infer that leucine has an influence on the metabolic and contractile type, as well as on muscle regeneration capacity. This potentially explains the fact that even without a muscle CSA, leucine was able to improve the muscle contraction parameters.

Although this present study was focused on the effects of leucine in improving muscle function and mass, the effect of leucine in adipose tissue needs to be highlighted. The white adipose tissue (WAT), as well as skeletal muscle, is usually depleted with cachexia. Adipose tissue spoliation is an important contributor to cachexia, since WAT synthesises many pro-inflammatory cytokines, contributing to systemic inflammation [41]. Moreover, some evidence has shown that WAT alterations precede muscle wasting [42,43]. The present study yielded interesting findings, i.e., that leucine supplementation ameliorated the perirenal adipose tissue mass in cancer cachexia animals. The molecular mechanisms of how leucine could act as a fat mass protective molecule in the context of cancer cachexia need further investigation.

Although the results presented here came from preclinical studies using an experimental model of cancer cachexia (Walker 256), this study gave rise to important findings that would be difficult to obtain through clinical trials. In the meantime, it is important to note some limitations in the present study. We carried out all the evaluations before the tumour injection and at the endpoint, where cachexia is evident and almost terminal. Considering that anorexia sometimes started on the 14th day of tumour growth, performing assessments also at this point would add some specific data about the cachexia stage. Also, leucine-rich diet administration was initiated on the same day as tumour inoculation, equating to the time of diagnosis for a patient; however, this could be a limitation in terms of a translational value of the results. Future studies should be considered in starting the diet administration at different points of tumour evolution as a treatment outcome. It is also important to highlight that new studies should investigate how leucine improves muscle function, evaluating both muscle and neural function and, specifically, the neuromuscular junction, to better understand the benefits of leucine. Also, considering that cachexia is a complex and multifactorial syndrome, a multimodal (e.g., a combination of nutritional scheme and physical exercise) treatment would be more efficient against the deleterious effects of cancer cachexia.

Summing up, here, we have presented some specific benefits of nutritional supplementation with leucine in a preclinical model of cancer cachexia. The leucine-rich diet improved body, fat, and muscle mass, which would be relevant considering the host′s responses to conventional treatment. Greater muscle mass was observed, even in the absence of any effect of leucine on muscle atrophy. Despite having no difference in muscle cross-section area, the nutritional scheme diminished the expression of some atrophy markers, such as MuRF-1 and 20S. However, in this experimental context, the leucine-rich diet was unable to improve muscle oxidative capacity. Finally, and most importantly, we found that a leucine-rich diet improved general behaviour (velocity and distance moved) and muscle strength, which led to a better quality of life for the study animals.

## Data Availability

The article detailing where the data support the results can be shared after the publication of the manuscript and can be found in https://osf.io/f54gs/.

## Supplementary Materials

The following are available online at https://www.mdpi.com/article/10.3390/cells10123272/s1, Figure S1: Catwalk functional (Walking test) parameters analyses in tumour-bearing groups, fed or not the leucine-rich diet, Figure S2: Evolution of behaviour and muscle force in tumour-bearing groups, fed or not the leucine-rich diet. Ecthovision behaviour parameters measured at night-time, Figure S3: Morphological parameters, Figure S4: Morphometric, histological and ultrastructural analyses of tibialis anterior muscle among the different experimental groups, Figure S5: Muscular gene and protein expression among the different experimental groups, Figure S6: Muscular gene and protein expression among the different experimental groups, Figure S7: Tibialis anterior muscle parameters.
