# Supplementary material for: Leucine-Rich Diet Improved Muscle Function in Cachectic Walker 256 Tumour-Bearing Wistar Rats
Source: Cells. 2021 Nov 23;10(12):3272. doi: 10.3390/cells10123272 (PMC8699792; doi:10.3390/cells10123272)
Supplement: Supplementary file 1 [file cells-10-03272-s001.zip › Supplementary Figure S1.pdf]

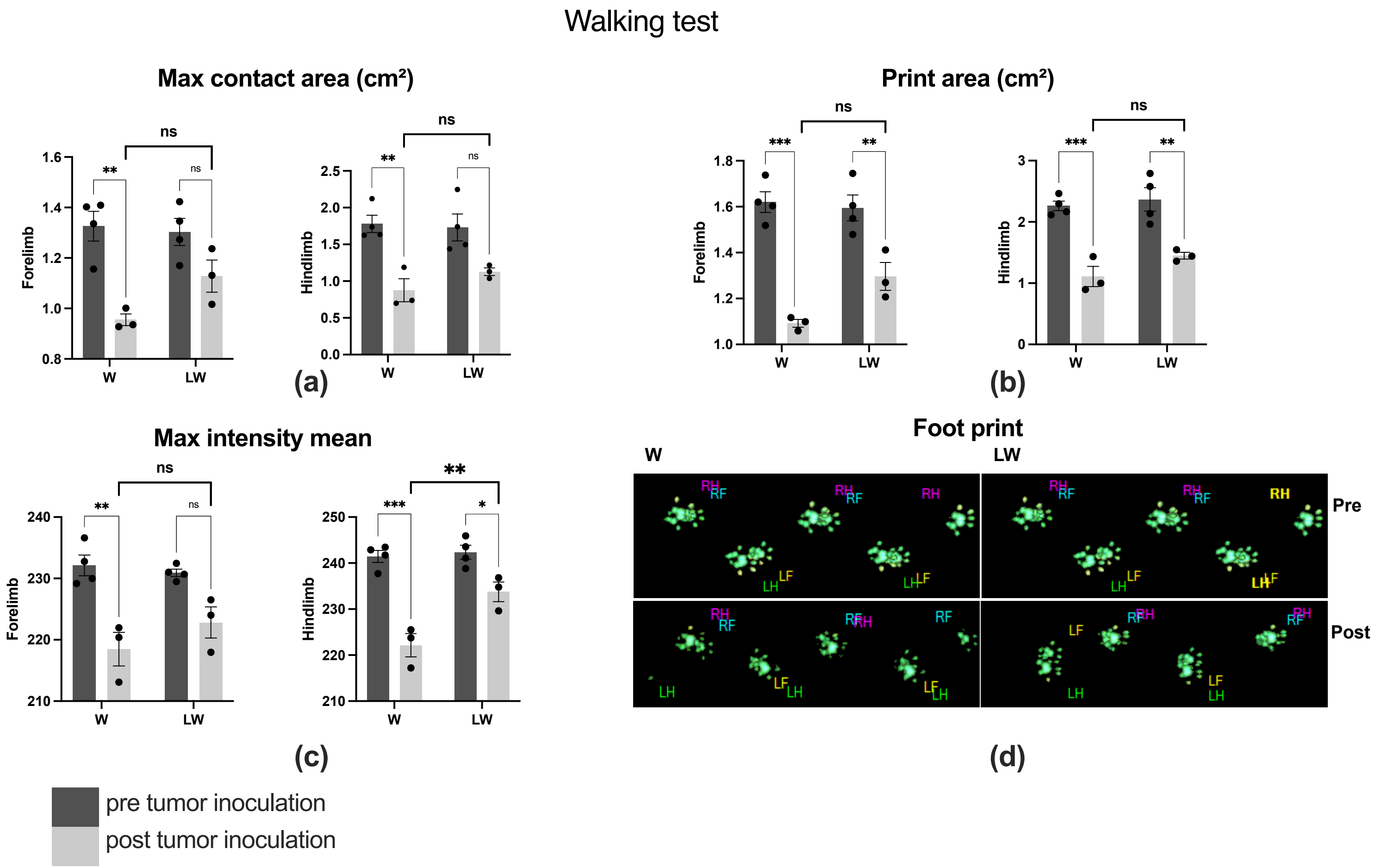
Walking test
Max contact area (cm²)
Print area (cm²)
Max intensity mean
Foot print
Forelimb
Hindlimb
W
LW
ns
**
***
*
(a)
(b)
(c)
(d)
RH
RF
LH
LF
Pre
Post
pre tumor inoculation
post tumor inoculation
