# Supplementary material for: Leucine-Rich Diet Improved Muscle Function in Cachectic Walker 256 Tumour-Bearing Wistar Rats
Source: Cells. 2021 Nov 23;10(12):3272. doi: 10.3390/cells10123272 (PMC8699792; doi:10.3390/cells10123272)
Supplement: Supplementary file 1 [file cells-10-03272-s001.zip › Supplementary figures legends.pdf]

Supplementary Figures Legends

Supplementary Figure 1. Catwalk functional (Walking test) parameters analyses in tumour-bearing groups, fed or not the leucine-rich diet. (a): Maximum contact area (cm2) in forelimb and hindlimb paws; (b): Print area (cm2) in forelimb and hindlimb paws; (c): Maximum intensity mean in forelimb and hindlimb paws and (d): A representative image of 2D walking pattern of both W and LW groups before and after (endpoint) tumour inoculation. Black bars represent the pre-tumour inoculation (health), and the grey bars represent the post-tumour inoculation (endpoint). The analysis was made using the same rats pre and post tumour evolution (*N*=4 for the pre tumour inoculation moment and *N*=3 for the post tumour inoculation moment, on the 18th day). For details, see the Methods section. RF – right front; LF – left front; RH – right hind; LH – left hind. The average forepaws (RF and LF) was considered forelimb, and the average hind paws (RH and LH) were considered hindlimb. Graphics represent mean ± SEM. *P<0.05, **P<0.01, ***P<0.001 and ns= not significant by ANOVA followed by Tukey's test.

Supplementary Figure 2. Evolution of behaviour and muscle force in tumour-bearing groups, fed or not the leucine-rich diet. Ecthovision behaviour parameters measured at night-time. (a) Time moving, (b) Velocity and (c) Distance moved. Black bars represent the pre-tumour inoculation, and the grey bars represent the post-tumour inoculation (endpoint). N = 6 animals per group. *P<0.05, **P<0.01, ***P<0.001 and ns= not significant by ANOVA followed by Tukey's test. Strength test measurement. (d): Strength (g) normalised by tibia length (mm) measured at endpoint moment, N = 4 animals per group. For details, see the Methods section. Graphics represent mean ± SEM. *P<0.05, ***P<0.001 and ns= not significant by ANOVA followed by Tukey's test.

Supplementary Figure 3. Morphological parameters. (a) Initial body weight (g); (b) Daily food intake (g) during the experimentation protocol; (c). Delta body weight (g) for non-tumour bearing groups (C and L; calculated as final body weight-initial body weight) and tumour-bearing groups (W and LW; calculated as (final body weight-tumour weight)-initial body weight); (d) Tibia length (mm); (e) Tumor weight (normalised by the respective tibia length); (f) White adipose tissue mass (perirenal) (normalised by the respective tibia length); (g) Spleen mass (normalised by the respective tibia length); (h) Serum IL6 concentration (pg/mL); (i) Serum TNF-α concentration (pg/mL). For details, see the Methods section. N = minimum of 6 animals per group. Graphics represent mean ± SEM. *P<0.05, **P<0.01, and ns= not significant by ANOVA followed by Tukey's test.

Supplementary Figure 4. Morphometric, histological and ultrastructural analyses of tibialis anterior muscle among the different experimental groups. (a) Tibialis anterior (TA) muscle mass (normalised by the respective tibia length). (b) Representative light microscopy of muscle tissue cross-section, stained by Hematoxylin Eosin, in C, L, W and LW groups (Scale bar: 60μm. Magnification 40x). (c) Measurements of myofibre cross-sectional area (μm^2). (d) Representative transmission electron microscopy of myofibre showing sarcomeric arrangement and mitochondria distribution at cytoplasm in C, L, W and LW groups (Scale bar: 2μm. Magnification at 10.000x). For details, see the Methods section. N = 6 animals per group. Graphics represent mean ± SEM. *P<0.05, **P<0.01, ***P<0.001 and ns= not significant by ANOVA followed by Tukey's test.

Supplementary Figure 5. Muscular gene and protein expression among the different experimental groups. Gene expression of (a) FoxO3, (b) IL6 and (c) ubiquitin. Protein expression. Western blot analysis images from (d) FoxO1, (e) MuRF-1, and (f) 20S expressions in tibialis anterior muscle biopsies. Bar graphs indicating western blot analysis representing values of band volume. GAPDH was the housekeeping protein. For details, see the Methods section. Graphics represent mean ± SEM. N = 6 animals per group. *P<0.05, ***P<0.001 and ns= not significant by ANOVA followed by Tukey's test.

Supplementary Figure 6. Muscular gene and protein expression among the different experimental groups. (a) Gene expression of citrate synthase and (b) (CS) cyclooxygenase (COX)5a. O2 consumption (ρmol O2 / s. mg tissue) compiled from the respiration traces comparing the (c) soleus and (d) EDL muscles biopsies from both tumour-bearing rats (W vs LW group). For details, see the Methods section. For gene expression *N* = 6 and for oxygen consumption *N* = 10–12, at least ten independent experiments. Graphics represent mean ± SEM. *P<0.05, ***P<0.001 and ns= not significant by ANOVA followed by Tukey's test.

Supplementary Figure 7. Tibialis anterior muscle parameters. (a) Tibialis anterior (TA) muscle mass (g); (b) Tibialis anterior (TA) muscle mass (normalised by the body weight); (c) Tibialis anterior (TA) muscle total protein concentration (μg/μL). For details, see the Methods section. N = minimum of 6 animals per group. Graphics represent mean ± SEM. *P<0.05 by t-test.
