# Supplementary figures and images for: Leucine-Rich Diet Improved Muscle Function in Cachectic Walker 256 Tumour-Bearing Wistar Rats
Source: Cells. 2021 Nov 23;10(12):3272. doi: 10.3390/cells10123272 (PMC8699792; doi:10.3390/cells10123272)

## Behavior test

## Strength test

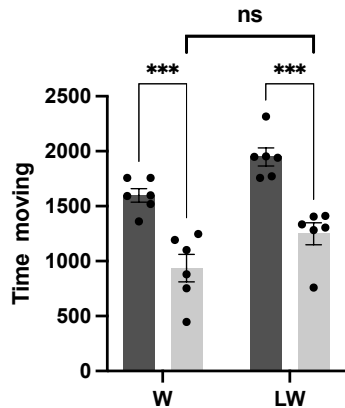

(a)

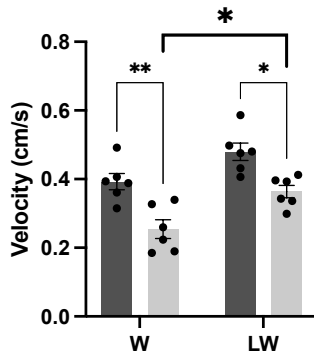

(b)

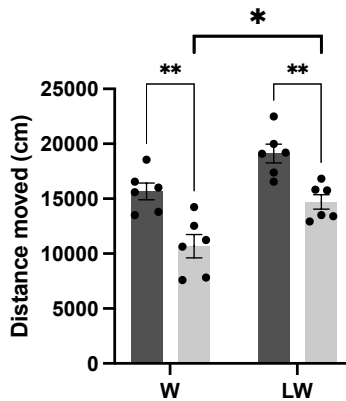

(c)

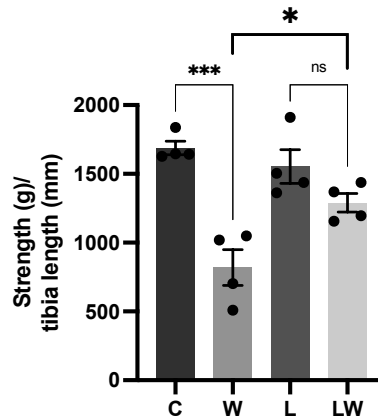

(d)

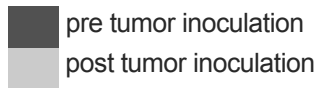

Supplement: Supplementary file 1 [file cells-10-03272-s001.zip › Supplementary Figure S2.pdf]

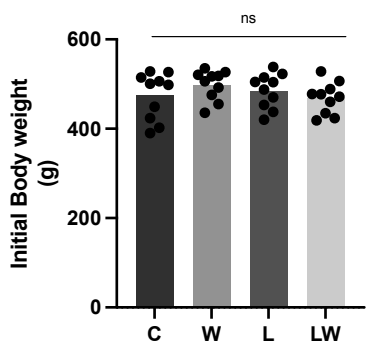

(a)

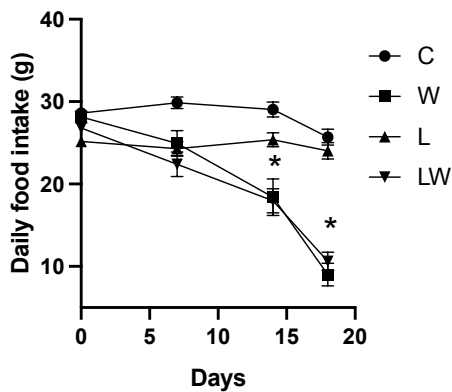

(b)

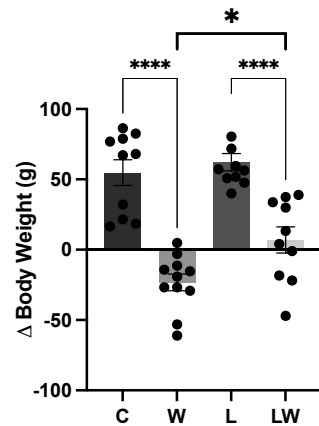

(c)

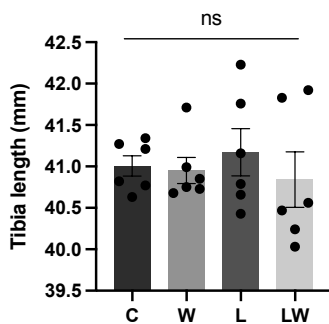

(d)

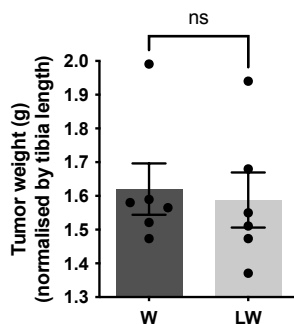

(e)

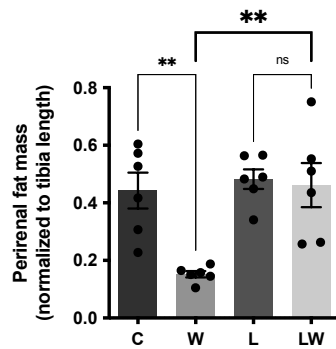

(f)

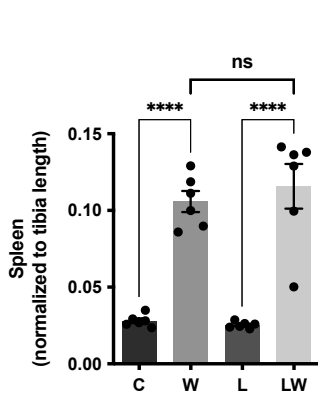

(g)

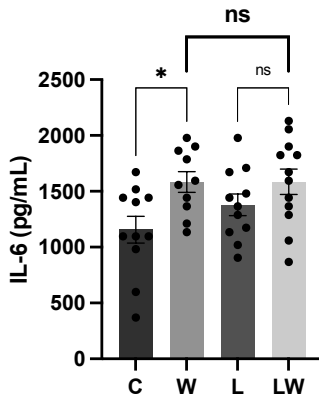

(h)

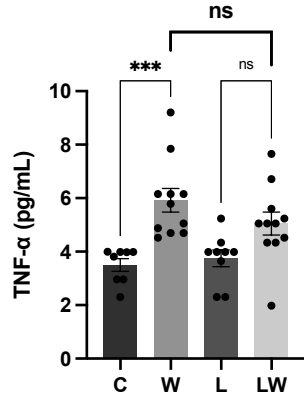

(i)

Supplement: Supplementary file 1 [file cells-10-03272-s001.zip › Supplementary Figure S3.pdf]

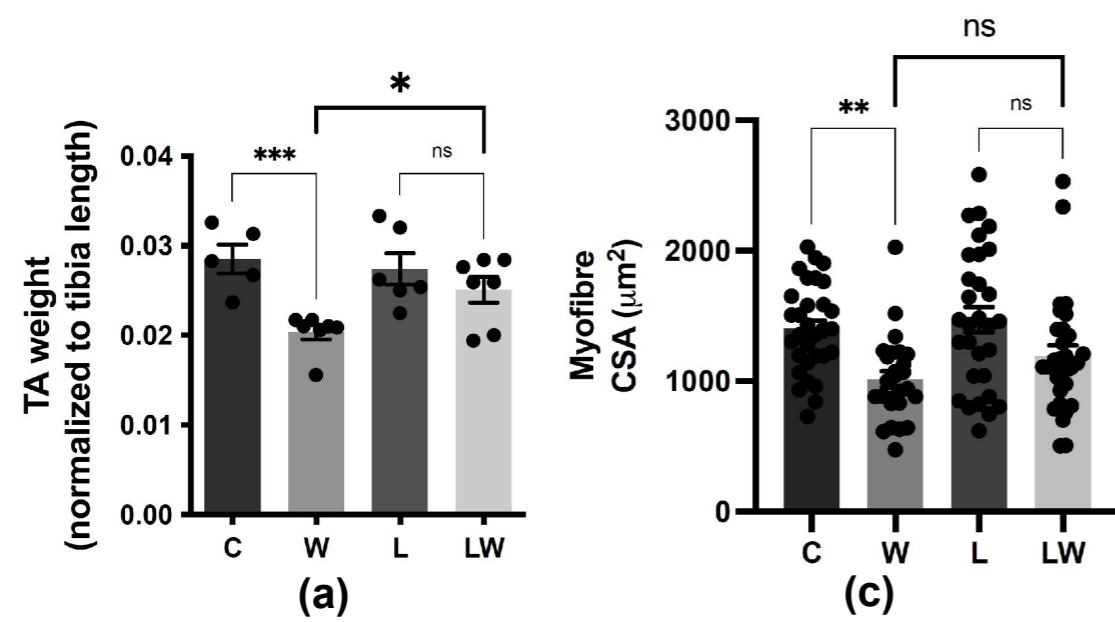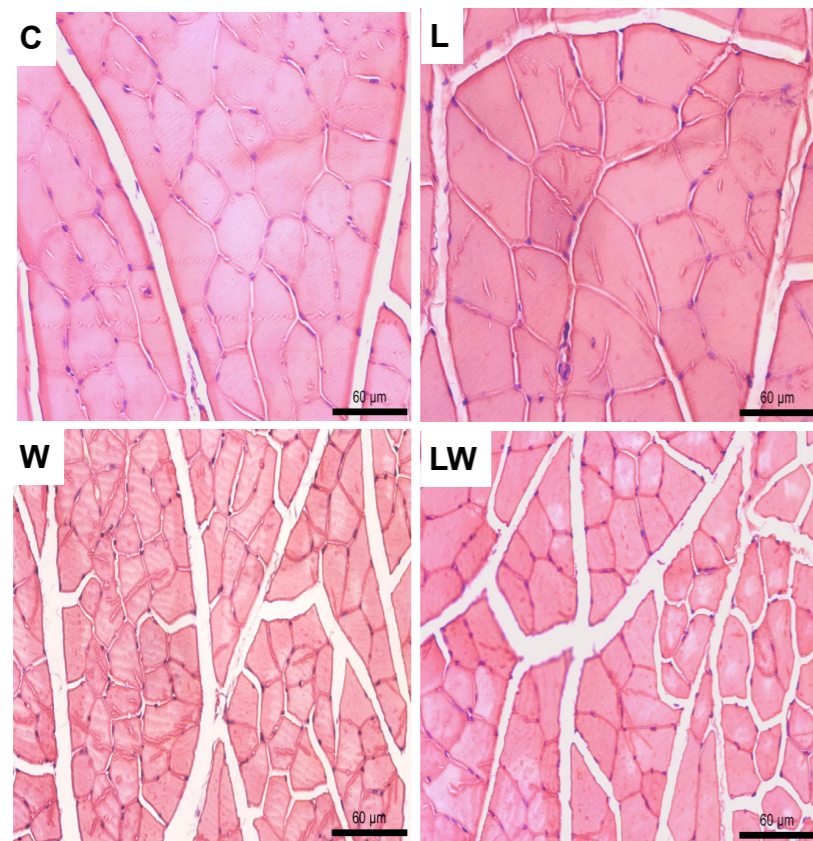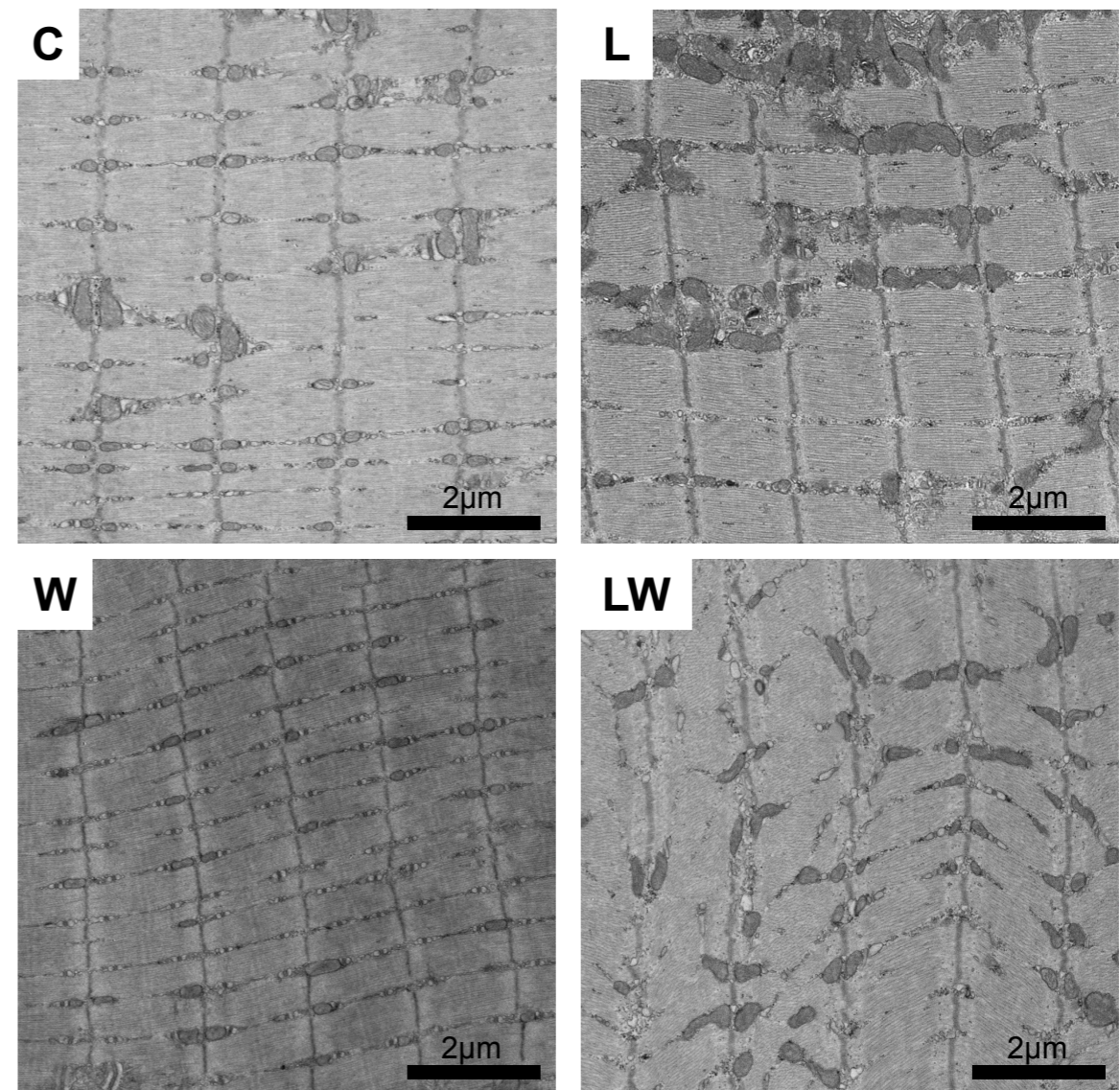

**(d)**

Supplement: Supplementary file 1 [file cells-10-03272-s001.zip › Supplementary Figure S4.pdf]

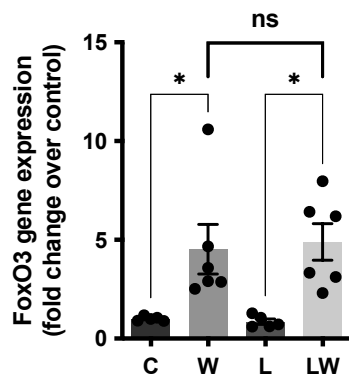

(a)

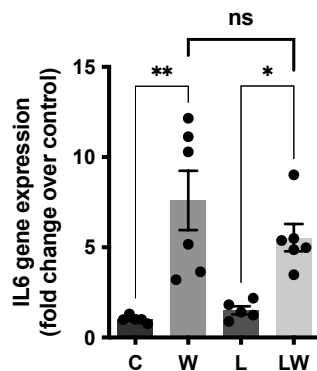

(b)

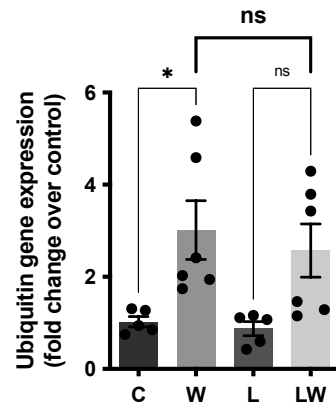

(c)

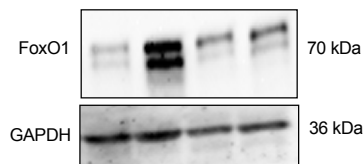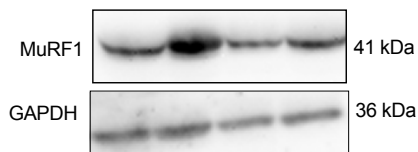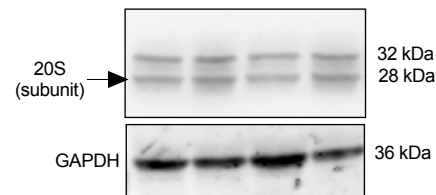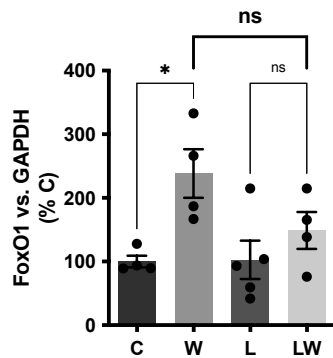

(d)

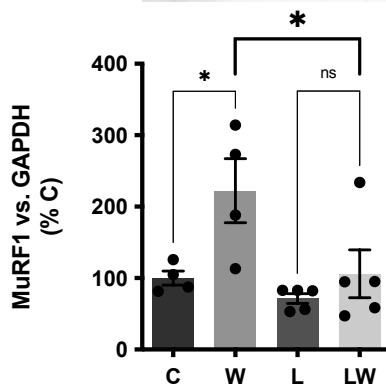

(e)

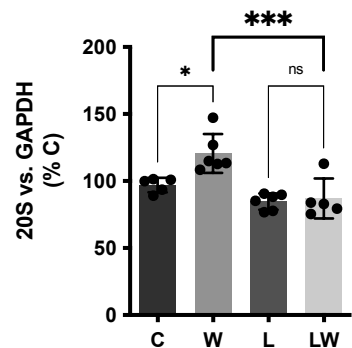

(f)

Supplement: Supplementary file 1 [file cells-10-03272-s001.zip › Supplementary Figure S5.pdf]

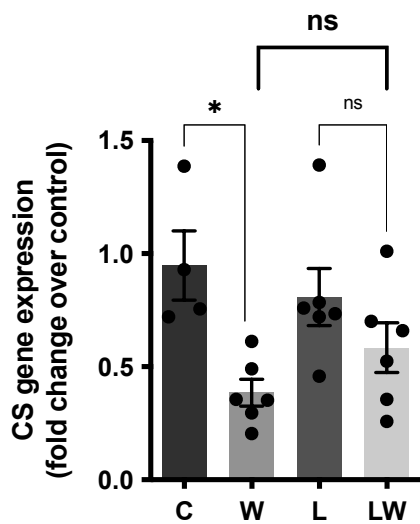

(a)

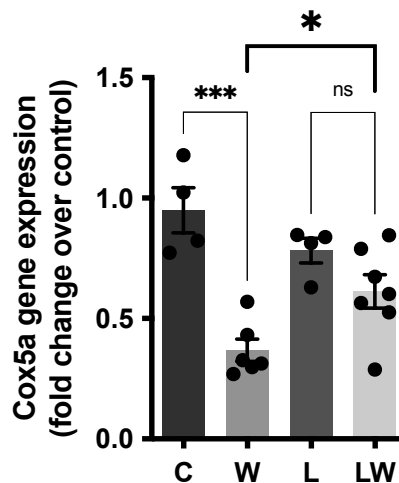

(b)

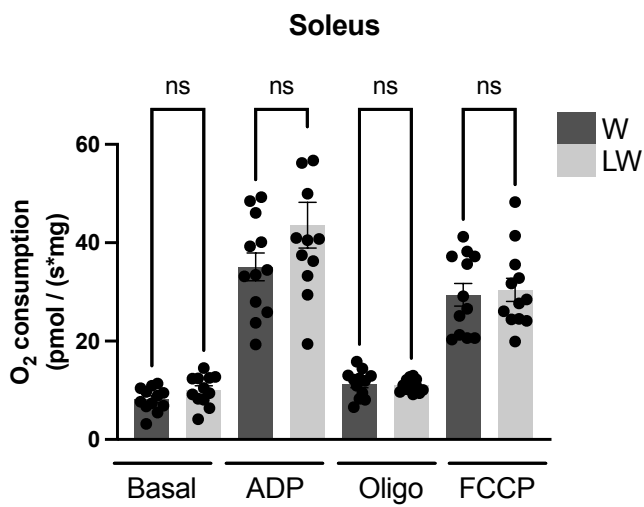

(c)

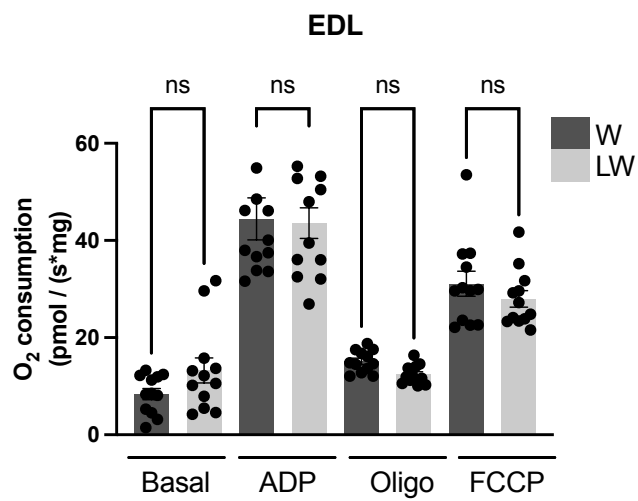

(d)

Supplement: Supplementary file 1 [file cells-10-03272-s001.zip › Supplementary Figure S6.pdf]

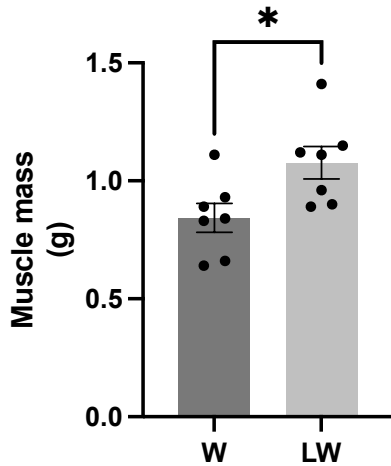

(a)

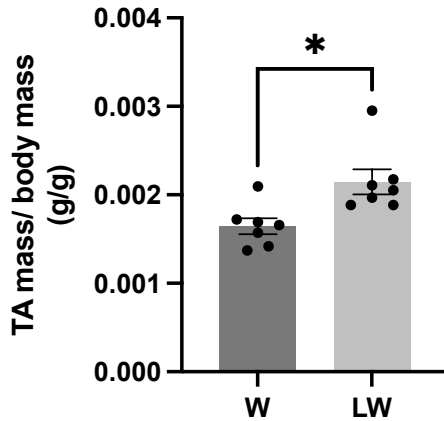

(b)

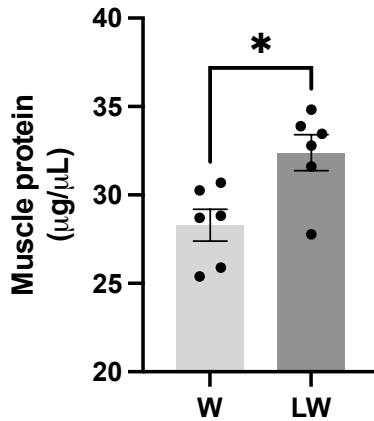

(c)

Supplement: Supplementary file 1 [file cells-10-03272-s001.zip › Supplementary Figure S7.pdf]
